# Cardiac-to-Bronchial Fistula in Hepatocellular Carcinoma: A Case Report

**DOI:** 10.3390/medicina60060982

**Published:** 2024-06-14

**Authors:** Hung-Hsu Chen, Yu-Hung Lai, Chin-Chu Wu, Wen-Pei Hsieh

**Affiliations:** Department of Radiology, Shin Kong Wu Ho-Su Memorial Hospital, No. 95, Wenchang Rd., Shilin Dist., Taipei 111, Taiwan; asahioncloud@gmail.com (H.-H.C.);

**Keywords:** hepatocellular carcinoma, diaphragmatic injury, liver abscess, radiotherapy, fistula, air emboli

## Abstract

Hepatocellular carcinoma (HCC) stands as a significant contributor to cancer-related mortality globally. While the acute and often fatal manifestations of locally advanced HCC primarily present within the abdomen, it is crucial to recognize that the respiratory and circulatory systems can also fall victim due to the liver’s unique anatomical position within the body. Here, we present the case of a 63-year-old male recently diagnosed with locally advanced HCC with vascular invasion. Shortly after receiving target therapy and focal radiotherapy, the patient developed repeated secondary infections and a persistent diaphragmatic defect. As the necrotic tissue invaded the pleural space, subsequent tumor-to-bronchial and tumor-to-cardiac fistulas emerged, resulting in an abnormal connection between the respiratory and cardiovascular systems, leading to massive air emboli in circulation. This report highlights the risk of supradiaphragmatic complications in HCC patients with post-treatment secondary infections, particularly in patients predisposed to developing diaphragmatic defects.

## 1. Introduction

Liver cancer poses a significant health burden worldwide, particularly in Asia, where it is one of the most prevalent cancers and the second leading cause of cancer-related death [1]. Hepatocellular carcinoma (HCC) predominates among these cases, primarily attributed to endemic hepatitis B virus (HBV) infection.

Although the clinical course of HCC varies by etiology and patient conditions, mortality primarily arises from three factors: tumor progression, liver failure, and tumor rupture [2]. While liver failure is a significant concern, particularly in patients with underlying cirrhosis, tumor progression stands out as the leading cause of mortality in most cases. Tumor progression is characterized by the spread of cancer cells, leading to the aggressive invasion of nearby organs and blood vessels, as well as distant metastases.

Tumor rupture, which can occur spontaneously or due to tumor necrosis following treatment, also leads to severe bleeding and life-threatening complications. Although improved surveillance and the early detection of HCC have decreased the incidence and mortality of tumor rupture [3], patients with certain characteristics, such as tumors protruding from Glisson’s capsule, those on free liver surfaces, and those with abnormal clotting function, remain at high risk [2].

Compared to the aforementioned factors, fatalities among HCC patients due to infection-related complications are relatively rare. Moreover, supradiaphragmatic involvement, affecting the pulmonary and cardiovascular systems, is seldom cited as a direct cause of death except in cases of distant metastasis. These complications, though rare, indicate potential risks in patients undergoing aggressive treatments and those with certain predisposing conditions. In this article, we describe a case of locally advanced HCC with post-treatment secondary infection, resulting in the development of a tumor-to-bronchial fistula and a tumor-to-cardiac fistula.

## 2. Case History

**Figure 1:**
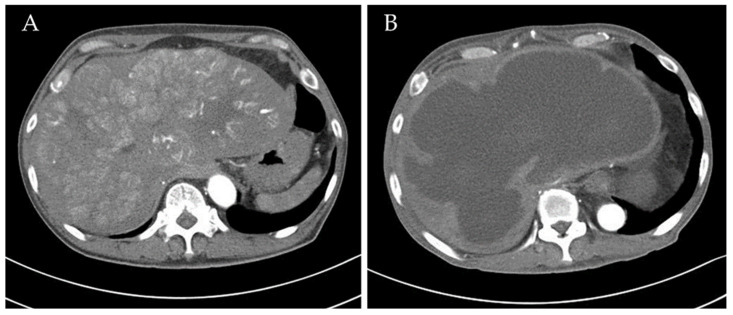
(**A**) The dynamic CT scan shows multiple hypervascular tumors attached to the liver dome during the late arterial phase. (**B**) The follow-up CT scan three months later reveals the disappearance of intratumoral arterial enhancement and some heterogeneous contents in most tumors, indicating tumor necrosis.

A 63-year-old male was incidentally diagnosed with HCC in April 2020. Initially, he presented with vague upper abdominal discomfort and weight loss. Ultrasound, followed by a dynamic CT scan, confirmed the diagnosis of multiple HCCs over the liver dome, staged at T4N0M0 with macrovascular invasion. The largest tumor measured 17 cm in diameter (Figure 1). Further investigations revealed the patient’s HBV carrier status and elevated Alpha Fetoprotein (AFP) levels.

**Figure 2:**
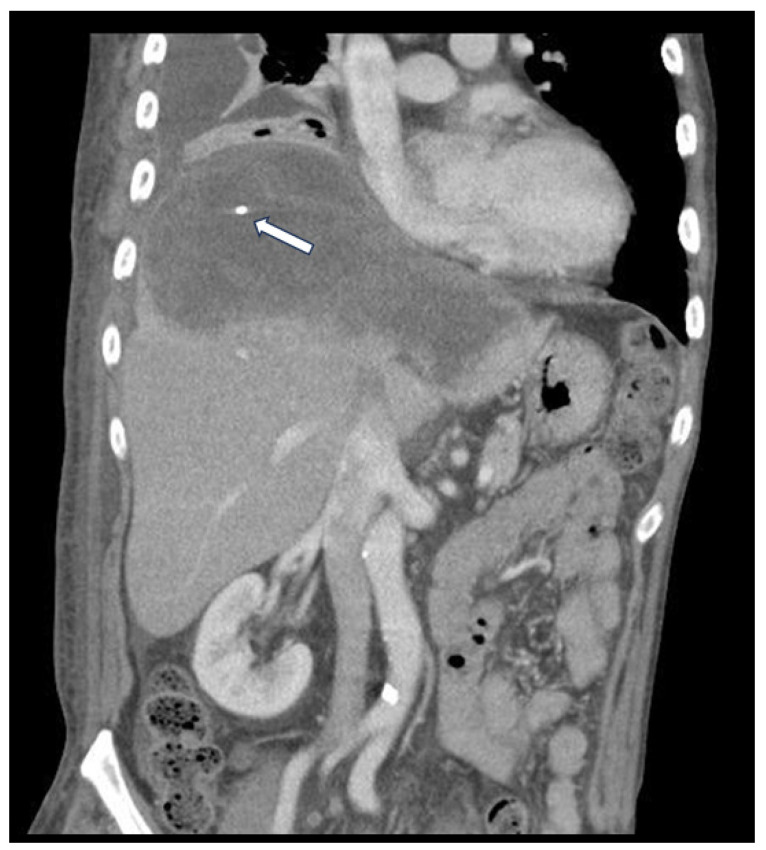
The contrast-enhanced CT scan reveals turbid debris scattered within the necrotic tumor, with ruptured contents extending through the diaphragmatic defect into the pleural space. The tip of the transhepatic drainage tube (white arrow) is visible.

Upon diagnosis, the patient underwent target therapy with lenvatinib as systemic treatment. The initial image follow-up after three months of treatment revealed a significant reduction in tumor vascularity, indicating a favorable treatment response (Figure 1). In addition to ongoing systemic treatment, the patient also underwent two cycles of proton beam therapy for local control in August and September 2020. However, in November 2020, the patient presented with aggravated abdominal discomfort and fever. A CT scan revealed turbid debris scattered within the necrotic tumor, with ruptured contents protruding through a diaphragmatic defect on the posterolateral aspect (Figure 2). Laboratory findings, including leukocytosis and elevated CRP levels, supported the diagnosis of secondary infection and liver abscess formation. The abscess culture later yielded positive results for Salmonella Typhi. Accordingly, the patient received appropriate antibiotic therapy and underwent transhepatic drainage.

**Figure 3:**
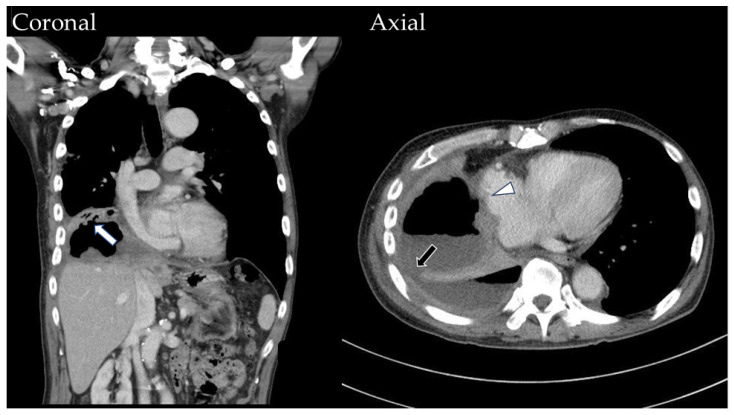
The contrast-enhanced CT scan reveals a tumor-to-bronchial fistula (white arrow) causing air accumulation on the coronal view. On the axial view, focal thinning of the cardiac wall (white arrowhead) and a persistent diaphragmatic defect (black arrow) over the posterolateral aspect are depicted.

In the following year, the patient was admitted for similar complaints two times. In August 2021, during yet another admission, the imaging study revealed a diaphragmatic defect over the posterolateral aspect, with necrotic tumor contents filling up the pleural space and forming a tumor-to-bronchial fistula (Figure 3). Accompanying lower lung atelectasis and pneumonia were also found and managed conservatively. 

The infection status persisted intermittently for another year. During this period, in addition to the mentioned treatments, we arranged thoracoscopic surgery for decortication and empyema removal. We also continued HCC systemic therapy under suitable clinical conditions. Unfortunately, we were unable to remove all the necrotic contents throughout the course, and no aggressive management was applied for the known fistulas and diaphragmatic defect.

**Figure 4:**
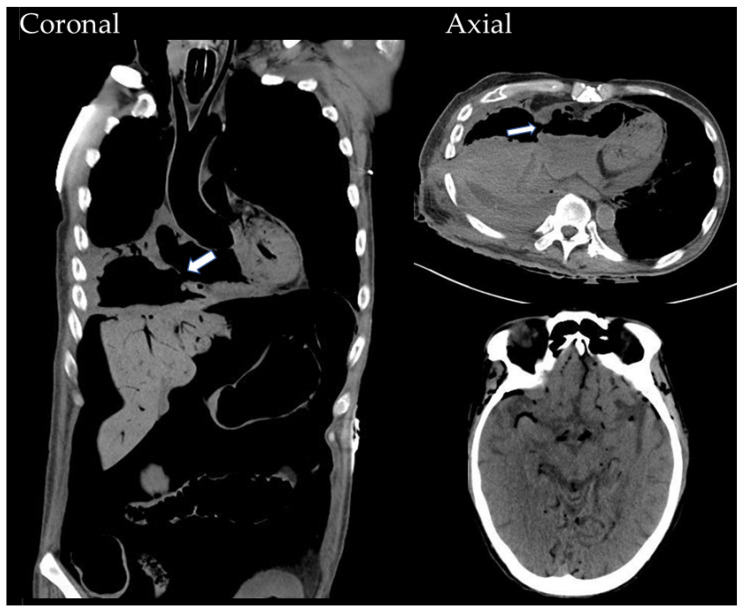
The emergent whole-body CT scan reveals a ruptured right atrium (white arrows) forming a bronchial-to-tumor-to-cardiac fistula. Massive air emboli are scattered in the cardiac chambers, great vessels, intracranial vessels, and pneumoperitoneum.

In October 2022, following a sudden onset of hemoptysis and loss of consciousness, the patient was sent to our emergency department. A whole-body CT scan revealed massive air emboli along the cardiovascular circulation, including the aorta, cardiac chambers, vena cava, intracranial vessels, and more. A newly developing tumor-to-right-atrium fistula connecting the cardiac chambers to the bronchus was identified (Figure 4). Despite the medical intervention provided, the patient expired soon afterward.

## 3. Discussion

This case reveals an unusual manifestation of post-treatment HCC. To understand the mechanism, we first need to review the tumor staging and corresponding treatment. According to the EASL Clinical Practice Guidelines and the Taiwan Liver Cancer Association HCC treatment guideline [4,5], locally advanced HCC with macrovascular invasion is unresectable and is treated with systemic therapy. Radiotherapy and transcatheter arterial chemoembolization (TACE) are also optional for local control. The present case initially received sorafenib for three months, resulting in the formation of a large necrotic volume and rupture of the tumor beyond the capsule into the subphrenic space.

Tumor rupture, as previously mentioned, is considered a dangerous complication for hypervascular tumors. The risk factors that were identified in this case before treatment included rapid growth, vascular invasion, and the peripheral or free-surface positioning of the tumor. Patients with larger tumors are also at risk of rupture due to increased pressure inside the tumor and vessels [6]. Invasive interventions such as transcatheter arterial embolization or hepatic resection may be needed to achieve hemostasis in patients. However, in the present case, the rapid response to treatment resulted in most of the tumor becoming devascularized, decreasing the bleeding tendency. In fact, there were no major adverse events during this period.

Secondly, extensive tumor necrosis led to the accumulation of dead tissue in the subphrenic space, triggering secondary infection and abscess formation in this case. In fact, liver abscesses in HCC patients are uncommon. They typically result from biliary obstruction, portal bacteremia, direct tissue invasion, systemic bacteremia, hepatic injury, or cryptogenic conditions. The relevant etiologies in HCC patients include malignancy-related biliary obstruction and contamination during procedures like radiofrequency ablation or TACE, which this patient did not undergo [7]. Regarding the treatment side effects, abscess formation after target therapy in HCC patients is extremely rare, with no known mechanism [8]. In this case, only the large tumor size and exceeded necrotic volume are worth noting as possible predisposing factors [9].

Special attention should be given to the pathogen; the abscess culture results revealed Salmonella Typhi, which is uncommon compared to more prevalent organisms like E. coli, Enterococcus, and Staphylococcus. Few reports have identified preexisting hepatobiliary diseases, including HCC, as a risk factor for Salmonella Typhi liver abscesses, however [10].

In the next step, to involve the area beyond the diaphragm, the infection must pass through it. Infected tissue can cause chronic septic injury, and the subphrenic position is crucial for prolonged tissue damage [11]. However, the diaphragm comprises a strong musculotendinous sheet that typically does not develop defects unless congenital or trauma-induced [12], suggesting other predisposing factors.

Current studies link diaphragmatic injury in HCC patients primarily to radiofrequency ablation, attributed to thermal damage or penetrant injury [13,14]. As for the treatment of the present case, while target therapy for HCC lacks known complications regarding diaphragmatic injury, radiotherapy has been documented to induce tissue damage and even lead to fistulas and perforations [15]. Radiation-induced complications have two phases: acute and chronic toxicities [16]. Acute toxicity results from damage to mucosal cells, while chronic toxicity involves vascular and connective tissue injury, often appearing months to years after radiotherapy completion. The present case of diaphragmatic perforation aligns with the potential chronic radiation-induced injury concerning exposure range and timeline.

Ultimately, infected tissue extended through the diaphragm into the pleural space, invading the nearby bronchus and forming a tumor-to-bronchial fistula. Our images show progressive air accumulation, causing elevated intratumoral pressure (Figure 3). This barotrauma, combined with chronic septic and radiation injuries, breached the cardiac wall, connecting the bronchus to the right atrium.

**Figure 5:**
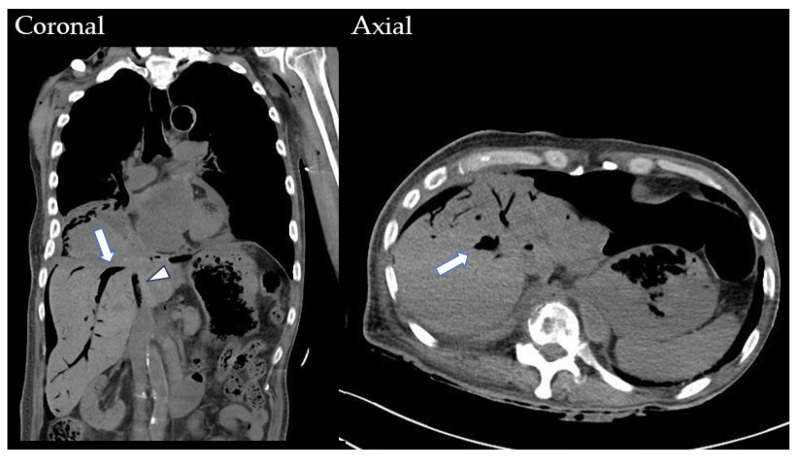
The same CT scan as shown in Figure 4 with different slices revealing air accumulation in the hepatic venous system (white arrow) with a claw-shaped appearance on the axial view. Air in the IVC is also visible (white arrowhead).

A noteworthy observation in the final CT scan is that as the air emboli traverse the circulatory system, the air accumulation in the hepatic venous system presents a distinctive appearance, with it extending outward from the liver dome, delineating the claw-shaped hepatic vein (Figure 5). This differs from the typical pattern observed in pneumobilia and pneumatosis portalis, which emanate from or converge around the hepatic hilum.

To our understanding, this represents the first documented case of such a manifestation of HCC-related fatality. In our assessment, tumor progression played a minor role, as evidenced by the absence of newly found enhanced tumor invasion during the follow-up. While there is no universally aligned management approach, we propose considering more aggressive surgical intervention to prevent chronic injury propagation. Repairing the diaphragmatic defect seems crucial to avoid supradiaphragmatic involvement. Various repair materials are available, including intercostal muscle, pericardial fat, omentum, synthetic mesh, or primary closure [17,18]. In cases where the diaphragm is irreparable, alternative interventions like bronchoscopic sealing or somatostatin analogues may be effective in closing the connection between the bronchus and tumors, although more evidence is needed [17].

## 4. Conclusions

This case report highlights the complex factors contributing to complications in HCC patients. While tumor rupture and even the formation of liver abscesses are documented, the development of fistulas and subsequent air emboli in this case is rare. The potential role of radiation-induced injury in diaphragmatic perforation underscores the need for more proactive monitoring and modifying the treatment plan accordingly. Aggressive surgical interventions may be necessary to prevent the extension of chronic injury. Also, further research is needed to determine management strategies.

## Data Availability

No new data were created or analyzed in this study.
